# Thrombocytopenia and Grading of Esophageal Varices in Patients With Chronic Liver Disease

**DOI:** 10.7759/cureus.60826

**Published:** 2024-05-22

**Authors:** Muhammad Asad Abbas, Aamir Ali, Saad Bin Zafar, Adeel Ahmed, Muhammad Noman Qureshi, Khizra Hamid, Muhammad Irfan Jamil, Iqra Naeem

**Affiliations:** 1 General Medicine, Pak Emirates Military Hospital, Rawalpindi, PAK; 2 General Medicine, Nishtar Medical University, Multan, PAK; 3 Acute Medicine, Queen Elizabeth Hospital Birmingham, Birmingham, GBR; 4 Medicine and Surgery, King Edward Medical University, Lahore, PAK; 5 Medicine, Lahore General Hospital, Lahore, PAK; 6 Internal Medicine, Frimley Park Hospital, Camberley, PAK; 7 Internal Medicine, Evercare Hospital, Lahore, Lahore, PAK; 8 Nephrology, Lahore General Hospital, Lahore, PAK; 9 Psychiatry and Behavioral Sciences, Lahore General Hospital, Lahore, PAK

**Keywords:** hcv, hbv, esophageal varices, thrombocytopenia, chronic liver disease

## Abstract

Background

Chronic liver disease (CLD) is associated with a variety of consequences, including thrombocytopenia and esophageal varices, which significantly impact patient prognosis and management. Thrombocytopenia, frequently observed in patients with CLD, may correlate with the severity of esophageal varices, a critical complication leading to variceal bleeding.

Methodology

A cross-sectional study was carried out in the Department of Medicine and Gastroenterology, Pak Emirates Military Hospital, Rawalpindi, from October 2021 to March 2022. The study enrolled 94 patients, aged 18-70 years, diagnosed with CLD, regardless of the cause. These patients were categorized into four groups based on platelet count: <50,000/uL, 50,000-99,999/uL, 100,000-150,000/uL, and >150,000/uL. Pearson's correlation was utilized to evaluate the association between the severity of thrombocytopenia and the grading of esophageal varices.

Results

A total of 94 patients were enrolled in the study, with 53 (56.4%) males and 41 (43.6%) females. The mean age of patients was 51.06 ±11.09 years. Seventeen (18.1%) had no esophageal varices, 16 (17.0%) were diagnosed with Grade I varices, 35 (37.2%) with Grade II varices, and 26 (27.7%) had Grade III varices. Most patients without varices had a platelet count above 150 x 10^3^ (17, 18.1%). Conversely, most patients with Grade III varices (19, 20.2%) had platelet counts below 50 x 10^3^. Patients with no esophageal varices had a mean platelet count of 173.70 ± 37.48 x 10^3^. Among the patients, those with Grade III esophageal varices exhibited the lowest mean platelet count, recorded at 78.54 ± 24.14 x 10^3^. These findings indicate a statistically significant difference in mean platelet counts across the various esophageal varices grades (*P* = 0.000). There was an inverse correlation of platelet count with the grading of esophageal varices (*r *= -0.645, *P* < 0.000).

Conclusions

A negative correlation was observed between the platelet count and the grading of esophageal varices, implying that as the severity of esophageal varices increased, the platelet counts proportionally decreased.

## Introduction

Chronic liver disease (CLD) is a prevalent condition in South Asia, with a significant proportion of patients progressing to liver cirrhosis and experiencing associated complications such as portal hypertension. This condition plays a crucial role in the development of complications including ascites, encephalopathy, and variceal bleeding [1,2]. Esophageal varices are a severe and life-threatening complication of portal hypertension often seen in patients with CLD. They are present in about 45% of compensated cirrhosis and 80% of uncompensated cirrhosis with ascites [3]. The majority of patients with CLD present late and most of them have large varices on their first screening [4]. A pressing need exists to better understand the intricacies of such complications and their prognosis in patients diagnosed with CLD.

While endoscopy remains the preferred method for screening patients for variceal development, it is accompanied by several drawbacks including invasiveness, discomfort, and limited accessibility. Furthermore, patients with cirrhosis require regular surveillance endoscopy, which can significantly burden the patient and the healthcare system. Ideally, a screening test for varices should be uncomplicated, quick, consistent, and cost-effective [5].

Thrombocytopenia, defined as a platelet count less than 150,000/μL, is a common complication observed in patients with portal hypertension and is prevalent in both cirrhotic and non-cirrhotic conditions. This complication is reported in approximately 76% of patients diagnosed with cirrhosis, highlighting the significant association between portal hypertension and thrombocytopenia [6]. The pathogenesis of thrombocytopenia in CLD is a multifaceted process involving a decrease in thrombopoietin production, splenic sequestration of platelets, and myelosuppressive effects, all of which culminate in a significant reduction in platelet count [4,7].

This study aims to investigate the association between thrombocytopenia and the grading of esophageal varices in patients with CLD. Thrombocytopenia frequently occurs in individuals with CLD and has been proposed as a potential stratification tool to identify low-risk patients for variceal bleeding, potentially reducing the need for unnecessary endoscopic screening. Through this study, we aimed to provide evidence that may enhance clinical decision-making processes regarding the management of esophageal varices in CLD patients.

## Materials and methods

Following approval from the ethical review committee (IRB number A/28/EC/436/2022) dated April 1, 2022, a descriptive cross-sectional study was conducted in the Department of Medicine and Gastroenterology, Pak Emirates Military Hospital, Rawalpindi. The study was completed over six months, from October 2021 to March 2022. The sample size of 94 was calculated, considering the expected correlation coefficient of 0.285. The alpha and beta were set at 0.05 and 0.20, respectively [6]. The sampling method employed was non-probability consecutive sampling.

Inclusion criteria

After obtaining informed consent from patients or their relatives, patients of either gender, aged between 18 and 70 years, who were diagnosed with chronic liver disease regardless of its cause, were enrolled in the study. This diagnosis of CLD was made on clinical, laboratory, and radiological findings.

Exclusion criteria

The study excluded patients who tested positive for human immunodeficiency virus (HIV) antibodies, those diagnosed with hepatocellular carcinoma or portal vein thrombosis, individuals who had received treatment for bleeding esophageal varices (EVs) either surgically or endoscopically, patients who had received drugs for primary prophylaxis of variceal bleeding, and those with a known case of chronic kidney disease or any hematological disorder. Patients with a history of recent blood component transfusion or any medication affecting platelet count were also deemed ineligible for the study.

Data collection

All participants received assurance regarding the confidentiality of their personal information and clinical data. A standard data collection form served as a tool for recording all necessary information, including demographic data (age, gender), clinical information, and the etiology of the chronic liver disease. Blood samples were collected and tested for a complete blood count, liver function, serum albumin, prothrombin time, and viral markers (hepatitis B surface antigen, anti-hepatitis C virus [HCV] antibodies, and HIV antibodies). An automatic hematology analyzer (Sysmex XN-1000) determined the platelet count. Endoscopic examinations were performed by a senior gastroenterologist with a minimum of three years of experience in upper gastrointestinal endoscopy. The presence and grade of EVs were determined using the modified Paquet grading system employed to ascertain the existence and severity of EVs. In the system's context, Grade I corresponds to varices that barely extend beyond the mucosal level. For Grade II, the varices project up to one-third of the luminal diameter and persist even when air insufflation is applied, showing their inability to be compressed. Grade III represents a more severe case where the varices expand to cover up to 50% of the luminal diameter, reaching the point where they are in contact with each other. Patients were categorized into four groups based on the platelet count: Group 1 had a platelet count of <50,000/uL, Group 2 between 50,000-99,999/uL, Group 3 between 100,000-150,000/uL, and Group 4 had a platelet count >150,000/uL. All the collected data were systematically entered into a preformatted perform for subsequent statistical analysis.

Data were entered and analyzed using IBM SPSS Statistics for Windows, Version 25.0 (IBM Corp., Armonk, NY). The analysis of demographic, clinical, and laboratory data involved the use of descriptive statistics, which included calculations of frequencies and percentages for categorical data such as gender, etiology, Child-Turcotte-Pugh (CTP) class, varices grades, and the presence or absence of ascites and encephalopathy. Mean and standard deviations were computed for continuous variables. Then, a chi-square test was implemented to evaluate the relationship between platelet count and the categorical variables. The differences between means across various platelet count categories for continuous variables were assessed using the analysis of variance (ANOVA) tests, and further analysis compared the mean platelet counts across different EV grades using the same method. Finally, Pearson's correlation coefficient was used in a correlation analysis to measure the relationship between platelet count and other laboratory parameters in relation to the grading of EVs, with a *P*-value of < 0.05 serving as an indicator of the statistical significance of these correlations.

## Results

A total of 94 patients were enrolled in the study, with 53 (56.4%) males and 41 (43.6%) females. The distribution of platelet counts differed significantly between males and females, with more males having counts below 50 x 10^3^, according to a study (*P *= 0.029). The majority of the patients (60, 63.8%) had liver cirrhosis and HCV, followed by HBV, and platelet counts did not differ significantly among these groups (*P *= 0.635). According to the CTP class, the majority of patients (53, 56.4%) were in class B and had platelet counts above 150 x 10^3^, followed by class A (20, 21.3%) and class C (21, 22.3%). This difference in platelet count among CTP classes was statistically significant (*P *= 0.000). Most patients without varices had a platelet count above 150 x 10^3^ (17, 18.1%). Conversely, most patients with Grade III varices (19, 20.2%) had platelet counts below 50 x 10^3^. Patients with Grades I and II varices displayed platelet counts that fell between these extremes (*P* = 0.000). The results indicated no significant difference in age, albumin, International Normalized Ratio (INR), and bilirubin levels across the platelet count categories, but there was a significant difference in hemoglobin levels between the categories (*P* = 0.000) (Table 1).

**Table 1:** Demographic, clinical, and laboratory data (n = 94).

		Total	Platelet count (x10 ^3^/uL)				*P*-value
			>150	100-150	50-99	<50	
Gender	Male	53 (56.4%)	9 (9.6%)	21 (22.3%)	17 (18.1%)	6 (6.4%)	0.029
	Female	41 (43.6%)	15 (36.6%)	17 (41.5%)	9 (22.0%)	0 (0.0%)	
Etiology	Hepatitis C virus (HCV)	60 (63.8%)	11 (11.7%)	26 (27.7%)	19 (20.2%)	4 (4.3%)	0.635
	Hepatitis B virus (HBV)	29 (30.9%)	11 (11.7%)	9 (9.6%)	7 (7.4%)	2 (2.1%)	
	Both	3 (3.2%)	1 (1.1%)	2 (2.1%)	0 (0.0%)	0 (0.0%)	
	Unknown	2 (2.1%)	1 (1.1%)	1 (1.1%)	0 (0.0%)	0 (0.0%)	
Child-Turcotte-Pugh (CTP) class	A	20 (21.3%)	13 (13.8%)	6 (6.4%)	1 (1.1%)	0 (0.0%)	0.000
	B	53 (56.4%)	8 (8.5%)	20 (21.3%)	19 (20.2%)	6 (6.4%)	
	C	21 (22.3%)	3 (3.2%)	12 (12.8%)	6 (6.4%)	0 (0.0%)	
Grades of esophageal varices	No varices	17 (18.1%)	13 (13.8%)	3 (3.2%)	1 (1.1%)	0 (0.0%)	0.000
	I	16 (17.0%)	5 (5.3%)	11 (11.7%)	0 (0.0%)	0 (0.0%)	
	II	35 (37.2%)	6 (6.4%)	20 (21.3%)	6 (6.4%)	3 (3.2%)	
	III	26 (27.7%)	0 (0.0%)	4 (4.3%)	19 (20.2%)	3 (3.2%)	
Ascites	Present	38 (40.4%)	16 (17.0%)	11 (11.7%)	10 (10.6%)	1 (1.1%)	0.016
	Absent	56 (59.6%)	8 (8.5%)	27 (28.7%)	16 (17.0%)	5 (5.3%)	
Encephalopathy	Present	35 (37.2%)	7 (7.4%)	13 (13.8%)	13 (13.8%)	2 (2.1%)	0.445
	Absent	59 (62.8%)	17 (18.1%)	25 (26.6%)	13 (13.8%)	4 (4.3%)	
Age (years)		51.06 ± 11.09	46.54 ± 13.05	51.89 ± 10.20	53.46 ± 9.14	53.50 ± 13.17	0.125
Albumin (g/dL)		3.05 ± 0.7	3.32 ± 0.69	2.92 ± 0.82	2.97 ± 0.70	3.23 ± 0.41	0.175
International normalized ratio (INR)		1.2 ± 0.3	1.17 ± 0.23	1.21 ± 0.31	1.27 ± 0.37	1.23 ± 0.29	0.685
Bilirubin (mg/dL)		2.1 ± 2.8	1.20 ± 0.87	2.7 ± 3.43	1.98 ± 2.42	3.58 ± 4.12	0.117
Hemoglobin (g/dL)		8.3 ± 0.9	8.90 ± 0.88	8.47 ± 0.83	7.83 ± 0.73	8.05 ± 0.95	0.000
Total			24 (25.5%)	38 (40.4%)	26 (27.7%)	6 (6.4%)	

Table 2 reveals that patients with no esophageal varices had a mean platelet count of 173.70 ± 37.48 x 10^3^. Among the patients, those with Grade III esophageal varices exhibited the lowest mean platelet count, recorded at 78.54 ± 24.14 x 10^3^. These findings indicated a statistically significant difference in mean platelet counts across the various esophageal varices grades (*P* = 0.000).

**Table 2:** Mean platelet counts comparison with esophageal varices grades (n = 94).

Esophageal varices grade	Mean ± standard deviation	*P*-value
No varices	173.70 ± 37.48	0.000
Grade I	145.19 ± 28.31	
Grade II	125.20 ± 49.92	
Grade III	78.54 ± 24.14	

The study findings revealed that the grading of esophageal varices had a strong negative correlation with the platelet count, a moderate negative correlation with albumin and hemoglobin, and a weak negative correlation with INR. However, bilirubin showed a weak positive correlation with variceal grading (Table 3).

**Table 3:** Correlation between platelet count and other laboratory parameters with the grading of esophageal varices.

	Pearson’s correlation	*P*-value
Platelet count	-0.645	0.000
Albumin	-0.337	0.000
International normalized ratio (INR)	-0.082	0.433
Bilirubin	0.205	0.048
Hemoglobin	-0.440	0.000

## Discussion

The global burden of CLD and liver cirrhosis is substantial, leading to over two million and one million deaths, respectively, every year [8]. These conditions are particularly devastating in Pakistan, contributing significantly to hospital admissions and mortality rates. The country is grappling with such a high prevalence of CLD that it has been designated a *cirrhotic state* [9]. The major drivers of CLD in Pakistan are hepatitis C and hepatitis B, with an estimated seven million people affected by hepatitis C, second only to China [10]. In our study, HCV and HBV were the primary causes of CLD, affecting 63.8% and 30.9% of cases, respectively, with a co-infection rate of 3.2%. This aligns with the existing literature, such as the study by Abbasi et al., where HCV antibodies were present in 77.5% of patients, hepatitis B surface antigen (HBsAg) in 11.8%, and co-infection in 7.8% [11]. Our findings underscored the significant role of HCV and HBV in CLD in Pakistan.

One severe complication of CLD is variceal gastrointestinal bleeding, a consequence of portal hypertension. This life-threatening event significantly contributes to patient morbidity, mortality, and healthcare costs [12,13]. Variceal hemorrhage occurs in 25% to 35% of patients with cirrhosis and is responsible for 80% to 90% of bleeding episodes in this patient population [14]. To manage this risk, the American Association for the Study of Liver Diseases (AASLD) recommends routine endoscopic screening for varices in patients with cirrhosis. The proposed frequency is one to three years, depending on the presence of varices and the state of the liver disease (compensated or decompensated) [15]. Despite the clear benefits of such a strategy, it presents a considerable strain on endoscopy units and places a significant financial burden on patients.

To mitigate these challenges, alternative screening tools have been explored. Thrombocytopenia, a common manifestation in patients with advanced cirrhosis, is one such potential marker. Previous research suggests a reliable correlation between the severity of thrombocytopenia and the presence of varices in patients with CLD [6]. In this study, we further investigated this relationship, particularly focusing on the ability of thrombocytopenia to predict the grade of EVs. 

In the present investigation, we observed a heterogeneous distribution of ages within our patient population, ranging from 18 to 70 years. The mean age of patients diagnosed with liver cirrhosis, irrespective of its root cause or the manifestation of EVs, was calculated to be 51.06 ± 11.09 years. This finding is in congruence with previous studies conducted by Abbasi et al. and Uong et al., which reported similar age distribution among their patient cohorts [11,16]. 

The current study revealed that a majority of patients without varices (60, 63.8%) had a platelet count >150 (x 10^3^/uL). However, with the escalation in the severity of EVs, a corresponding decrease in platelet count became evident. The highest percentage of patients (13, 13.8%) who did not have varices registered a platelet count >150 (x10^3^/uL). Conversely, no patients (0%) with grade III varices were observed to have a platelet count within this range. An inverse correlation emerged distinctly when considering the prevalence of severe thrombocytopenia (<50 x 10^3^/uL), which was the highest in patients with grade III varices (19, 20.2%). These observations echo the findings of Priyadarshi et al. [17]. They reported that patients with higher platelet counts (>150 and >200) primarily had no or grade I varices. Conversely, those with lower platelet counts (<50 and 50-99) were more inclined toward higher grades of varices [17]. Further support for our findings came from the study by Abbasi et al. For instance, in the study by Abbasi et al., within the platelet count ≤20 x 10^3^/uL group, a substantial 66.66% of patients had grade IV varices, the most severe grade. In the 21-50 x 10^3^/uL platelet count range, half of the patients (50%) were observed with grade IV varices. This trend continued even in the higher platelet count categories: in the 50-99 x 10^3^/uL group, 30.35% of patients had grade IV varices, while in the 100-150 x 10^3^/uL range, 15.38% of patients were diagnosed with grade IV varices. These findings further underscore the inverse relationship our study identified between platelet count and the severity of EVs [11].

This research demonstrated a strong negative correlation between mean platelet count and EV severity, aligning with the findings of Nadeem et al. Our data showed that patients with no EVs had a higher mean platelet count of 173.70 ± 37.48 x 10^3^/uL, whereas the lowest count of 78.54 ± 24.14 x 10^3^/uL was found in those with grade III EVs. Nadeem et al.'s study also revealed a similar trend: as the variceal grade increased from I to IV, the mean platelet counts significantly decreased from approximately 213 x 10^3^/mm^3^ to around 21 x 10^3^/mm^3^ [18]. Consequently, all studies reaffirmed the inverse relationship between platelet count and EV severity.

Both our research and the study by Abbasi et al. highlighted the negative correlation between platelet count and EVs. Our data showed a stronger correlation (-0.645 vs. -0.321), suggesting lower platelet counts could more significantly indicate higher grades of EVs. Similarly, lower hemoglobin levels were associated with EVs in both studies, with our findings showing a more significant correlation (-0.440 vs. -0.195). However, contrasts were found with bilirubin and albumin. Our results showed a weak positive correlation with bilirubin (0.205) and a notable negative correlation with albumin (-0.337), unlike Abbasi et al.'s insignificant correlations (bilirubin: -0.086, albumin: -0.036), indicating divergent roles of these parameters in the presence of EVs [11]. Similarly, Nadeem et al. demonstrated a remarkably strong negative correlation (*r *= -0.783) [18]. It's notable that most studies, including ours, showed a negative correlation, reaffirming that platelet counts are a valuable indicator of predicting higher EVs.

Our study does have several limitations that need to be addressed. First, despite the high prevalence of CLD, our study was based on a small sample size, which might not accurately represented the overall population with this condition. Second, this study was single-centered, limiting the geographic diversity of the sample and potentially introducing bias. The third limitation was that we did not establish a cutoff for platelet count to evaluate sensitivity and specificity, which might have impacted the robustness of our correlation findings. Finally, our study lacked variety in the cases of CLD, as most of our patients had chronic viral hepatitis. This lack of variety could limit the generalizability of our findings to other types of CLD. Future studies with larger and more diverse samples are needed to validate and expand upon our findings.

## Conclusions

In conclusion, our study unequivocally demonstrates a significant association between platelet count and grades of EVs. A clear negative correlation was observed between the platelet count and the grading of EVs, implying that as the severity of EVs increased, the platelet counts proportionally decreased. This relationship implies that the platelet count may serve as a valuable prognostic indicator for the severity of EVs in this patient population.
